# Supplementary material for: HIVprotI: an integrated web based platform for prediction and design of HIV proteins inhibitors
Source: J Cheminform. 2018 Mar 9;10:12. doi: 10.1186/s13321-018-0266-y (PMC5845081; doi:10.1186/s13321-018-0266-y)
Supplement: Supplementary file 2 — Additional file 2. Source code of HIVProtI web server. [file 13321_2018_266_MOESM2_ESM.zip › HIVprotI_Source-code/hivprotI/weka-3-6-6/doc/weka/gui/SortedTableModel.html]

SortedTableModel


---


|  |  |  |
| --- | --- | --- |
| **Overview** \| **Package** \| **Class** \| **Tree** \| **Deprecated** \| **Index** \| **Help** \| **Weka's home** | |  |
| **PREV CLASS**   **NEXT CLASS** | **FRAMES**    **NO FRAMES**     **All Classes** |  |
| SUMMARY: NESTED \| FIELD \| CONSTR \| METHOD | DETAIL: FIELD \| CONSTR \| METHOD |  |


---


## weka.gui Class SortedTableModel

```
java.lang.Object
  javax.swing.table.AbstractTableModel
      weka.gui.SortedTableModel
```

**All Implemented Interfaces:**: java.io.Serializable, java.util.EventListener, javax.swing.event.TableModelListener, javax.swing.table.TableModel

**Direct Known Subclasses:**: ArffSortedTableModel

---

``` public class SortedTableModel extends javax.swing.table.AbstractTableModel implements javax.swing.event.TableModelListener ```

Represents a TableModel with sorting functionality.

**Version:**
:   $Revision: 7059 $

**Author:**
:   FracPete (fracpete at waikato dot ac dot nz)

**See Also:**: Serialized Form

---

| **Nested Class Summary** | |
| --- | --- |
| `static class` | `SortedTableModel.SortContainer`             Helper class for sorting the columns. |


**Constructor Summary**

`SortedTableModel()`
  
          initializes with no model

`SortedTableModel(javax.swing.table.TableModel model)`
  
          initializes with the given model


| **Method Summary** | |
| --- | --- |
| `void` | `addMouseListenerToHeader(javax.swing.JTable table)`             Adds a mouselistener to the header: left-click on the header sorts in ascending manner, using shift-left-click in descending manner. |
| `int` | `getActualRow(int visibleRow)`             Returns the actual underlying row the given visible one represents. |
| `java.lang.Class` | `getColumnClass(int columnIndex)`             Returns the most specific superclass for all the cell values in the column. |
| `int` | `getColumnCount()`             Returns the number of columns in the model |
| `java.lang.String` | `getColumnName(int columnIndex)`             Returns the name of the column at columnIndex |
| `javax.swing.table.TableModel` | `getModel()`             returns the current model, can be null |
| `int` | `getRowCount()`             Returns the number of rows in the model. |
| `java.lang.Object` | `getValueAt(int rowIndex, int columnIndex)`             Returns the value for the cell at columnIndex and rowIndex. |
| `boolean` | `isCellEditable(int rowIndex, int columnIndex)`             Returns true if the cell at rowIndex and columnIndex is editable. |
| `boolean` | `isSorted()`             returns whether the table was sorted |
| `void` | `setModel(javax.swing.table.TableModel value)`             sets the model to use |
| `void` | `setValueAt(java.lang.Object aValue, int rowIndex, int columnIndex)`             Sets the value in the cell at columnIndex and rowIndex to aValue. |
| `void` | `sort(int columnIndex)`             sorts the table over the given column (ascending) |
| `void` | `sort(int columnIndex, boolean ascending)`             sorts the table over the given column, either ascending or descending |
| `void` | `tableChanged(javax.swing.event.TableModelEvent e)`             This fine grain notification tells listeners the exact range of cells, rows, or columns that changed. |

**Methods inherited from class javax.swing.table.AbstractTableModel**

`addTableModelListener, findColumn, fireTableCellUpdated, fireTableChanged, fireTableDataChanged, fireTableRowsDeleted, fireTableRowsInserted, fireTableRowsUpdated, fireTableStructureChanged, getListeners, getTableModelListeners, removeTableModelListener`

**Methods inherited from class java.lang.Object**

`equals, getClass, hashCode, notify, notifyAll, toString, wait, wait, wait`

**Constructor Detail**

### SortedTableModel

```
public SortedTableModel()
```

:   initializes with no model

---


### SortedTableModel

```
public SortedTableModel(javax.swing.table.TableModel model)
```

:   initializes with the given model

    **Parameters:**: `model` - the model to initialize the sorted model with


**Method Detail**

### setModel

```
public void setModel(javax.swing.table.TableModel value)
```

:   sets the model to use

    :   **Parameters:**: `value` - the model to use

---


### getModel

```
public javax.swing.table.TableModel getModel()
```

:   returns the current model, can be null

    :   **Returns:**: the current model

---


### isSorted

```
public boolean isSorted()
```

:   returns whether the table was sorted

    :   **Returns:**: true if the table was sorted

---


### getActualRow

```
public int getActualRow(int visibleRow)
```

:   Returns the actual underlying row the given visible one represents. Useful
    for retrieving "non-visual" data that is also stored in a TableModel.

    :   **Parameters:**: `visibleRow` - the displayed row to retrieve the original row for **Returns:**: the original row

---


### getColumnClass

```
public java.lang.Class getColumnClass(int columnIndex)
```

:   Returns the most specific superclass for all the cell values in the
    column.

    :   **Specified by:**: `getColumnClass` in interface `javax.swing.table.TableModel` **Overrides:**: `getColumnClass` in class `javax.swing.table.AbstractTableModel`
    :   **Parameters:**: `columnIndex` - the index of the column **Returns:**: the class of the specified column

---


### getColumnCount

```
public int getColumnCount()
```

:   Returns the number of columns in the model

    :   **Specified by:**: `getColumnCount` in interface `javax.swing.table.TableModel`
    :   **Returns:**: the number of columns in the model

---


### getColumnName

```
public java.lang.String getColumnName(int columnIndex)
```

:   Returns the name of the column at columnIndex

    :   **Specified by:**: `getColumnName` in interface `javax.swing.table.TableModel` **Overrides:**: `getColumnName` in class `javax.swing.table.AbstractTableModel`
    :   **Parameters:**: `columnIndex` - the column to retrieve the name for **Returns:**: the name of the specified column

---


### getRowCount

```
public int getRowCount()
```

:   Returns the number of rows in the model.

    :   **Specified by:**: `getRowCount` in interface `javax.swing.table.TableModel`
    :   **Returns:**: the number of rows in the model

---


### getValueAt

```
public java.lang.Object getValueAt(int rowIndex,
                                   int columnIndex)
```

:   Returns the value for the cell at columnIndex and rowIndex.

    :   **Specified by:**: `getValueAt` in interface `javax.swing.table.TableModel`
    :   **Parameters:**: `rowIndex` - the row: `columnIndex` - the column **Returns:**: the value of the sepcified cell

---


### isCellEditable

```
public boolean isCellEditable(int rowIndex,
                              int columnIndex)
```

:   Returns true if the cell at rowIndex and columnIndex is editable.

    :   **Specified by:**: `isCellEditable` in interface `javax.swing.table.TableModel` **Overrides:**: `isCellEditable` in class `javax.swing.table.AbstractTableModel`
    :   **Parameters:**: `rowIndex` - the row: `columnIndex` - the column **Returns:**: true if the cell is editable

---


### setValueAt

```
public void setValueAt(java.lang.Object aValue,
                       int rowIndex,
                       int columnIndex)
```

:   Sets the value in the cell at columnIndex and rowIndex to aValue.

    :   **Specified by:**: `setValueAt` in interface `javax.swing.table.TableModel` **Overrides:**: `setValueAt` in class `javax.swing.table.AbstractTableModel`
    :   **Parameters:**: `aValue` - the new value of the cell: `rowIndex` - the row: `columnIndex` - the column

---


### sort

```
public void sort(int columnIndex)
```

:   sorts the table over the given column (ascending)

    :   **Parameters:**: `columnIndex` - the column to sort over

---


### sort

```
public void sort(int columnIndex,
                 boolean ascending)
```

:   sorts the table over the given column, either ascending or descending

    :   **Parameters:**: `columnIndex` - the column to sort over: `ascending` - ascending if true, otherwise descending

---


### tableChanged

```
public void tableChanged(javax.swing.event.TableModelEvent e)
```

:   This fine grain notification tells listeners the exact range of cells,
    rows, or columns that changed.

    :   **Specified by:**: `tableChanged` in interface `javax.swing.event.TableModelListener`
    :   **Parameters:**: `e` - the event

---


### addMouseListenerToHeader

```
public void addMouseListenerToHeader(javax.swing.JTable table)
```

:   Adds a mouselistener to the header: left-click on the header sorts in
    ascending manner, using shift-left-click in descending manner.

    :   **Parameters:**: `table` - the table to add the listener to


---


|  |  |  |
| --- | --- | --- |
| **Overview** \| **Package** \| **Class** \| **Tree** \| **Deprecated** \| **Index** \| **Help** \| **Weka's home** | |  |
| **PREV CLASS**   **NEXT CLASS** | **FRAMES**    **NO FRAMES**     **All Classes** |  |
| SUMMARY: NESTED \| FIELD \| CONSTR \| METHOD | DETAIL: FIELD \| CONSTR \| METHOD |  |


---
